# Self-passivating (Re,Al)B_2_ coatings synthesized by magnetron sputtering

**DOI:** 10.1038/s41598-018-34042-1

**Published:** 2018-10-22

**Authors:** Pascal Bliem, Stanislav Mráz, Sandipan Sen, Oliver Hunold, Jochen M. Schneider

**Affiliations:** Materials Chemistry, RWTH Aachen University, Kopernikusstr. 10, D-52074 Aachen, Germany

## Abstract

(Re_0.67_Al_0.10_)B_2_ and (Re_0.74_Al_0.11_)B_2_ solid solution as well as Re_0.85_B_2_ thin films were deposited by hybrid RF-DC magnetron sputtering. X-ray diffraction (XRD) showed that all films exhibit the ReB_2_ (*P6*_3_*/mmc*) crystal structure. X-ray photoelectron spectroscopy (XPS) analyses performed on atmosphere exposed thin film surfaces suggest that ReB_2_ corrodes, consistent with literature, by forming perrhenic acid (HReO_4_) already after two days, while (Re_0.74_Al_0.11_)B_2_ forms a self-passivating Al-oxide layer preventing corrosion in a time period ≥ 60 days. Hence, it is evident that Al additions to ReB_2_ significantly increase the chemical stability during atmosphere exposure.

## Introduction

The increasing number of highly specialized industrial machining applications creates a demand for suitable new hard coating material systems. Commonly known superhard (*H* ≥ 40 GPa) materials such as diamond (up to 150 GPa), cubic BN (up to 80 GPa)^1^, B_6_O (up to 45 GPa)^2^, and cubic BC_2_N (up to 75 GPa)^3^, are all electrical insulators and can sometimes not be utilized, e.g. during machining of ferrous alloys with diamond-like coatings^4^ due to carbide formation. Borides are used as hard and wear-resistant coatings^5^, e.g. in Al machining due to low Al adhesion on the coated tool surface^6^. ReB_2_ (*P6*_3_*/mmc*), first synthesized in 1962 by La Placa *et al*.^7^, is metallic^8^ and was first suggested to exhibit a hardness above 40 GPa little more than a decade ago by Chung *et al*.^4^. Density functional theory calculations of ReB_2_^9–12^ show that B-B and Re-B bonds are short and highly directionally covalent, therefore strong, whereas Re-Re bonds are predominantly metallic.

Chung *et al*.^4^ reported a hardness of 48 GPa at 0.5 N indentation load which, however, decreased with increasing load down to 30 GPa at 5 N load. Other experimental studies report largely scattered values of measured hardness (*H* = 38(11) GPa^4,13–18^, notation: average value (standard deviation on the last significant digits)). One reason for this scattering can be the presence Re_7_B_3_ phase impurities which are often reported^17,19–22^ and which highlight the necessity of obtaining phase-pure samples for a reliable characterization. Despite several of the studies mentioned above have suggested that ReB_2_ may be a promising candidate for hard coating applications, only two studies have synthesized ReB_2_ thin films by pulsed laser deposition^15,18^. Experiments employing methods which can be used on large industrial scales, such as magnetron sputtering, are yet lacking.

Besides the need for further research on thin film synthesis, there is a necessity to evaluate the material’s chemical stability. Two studies^16,23^ reported formation of a viscous liquid layer on their samples exposed to air. Orlovskaya *et al*.^23^ hypothesized that on their mechanically milled powders, Re_2_O_7_ and B_2_O_3_ oxides react with water from air and form perrhenic acid (HReO_4_) and boric acid (H_3_BO_3_), respectively. Due to hygroscopicity of these acids, they may adsorb further water from air and continue to degrade the bulk material. The authors’ hypothesis was based on thermodynamic data and not proven experimentally. Very recently, Granados-Fitch *et al*.^24^ extended Orlovskaya *et al*.’s work by experimentally studying the reaction of mechanically milled powders in humid air over duration of 26 months. Theses ReB_2_ powders decomposed entirely into HReO_4_ (liquid), H_3_BO_3_, HBO_2_, and ReO_3_. The corrosive reaction is possibly accelerated by the catalytic activity of perrhenic acid^25,26^.

Alloying of Al may represent a method to counteract the potential corrosive reaction. The addition of Al to TiN thin films results in the formation of alumina upon oxidation and an improved oxidation resistance of the ternary TiAlN, compared to the binary TiN^27^. Even in borides with a small Al content, such as amorphous AlYB_14_, Al is preferentially oxidized^5^. Furthermore, Al is not only known for its oxygen affinity and stable Al_2_O_3_ oxide which is commonly used as a diffusion barrier^28^; it also forms a hexagonal diboride AlB_2_ (*P6/mmm*) (though not isostructural to ReB_2_ as B layers in AlB_2_ are flat and not puckered). It has been shown previously that alloying W, which also forms a hexagonal diboride WB_2_ (*P6*_3_*/mmc*) with both flat and puckered B layers, into ReB_2_ can yield in solid solutions with high hardness (up to 48 GPa)^29^. Hence, it is promising to investigate Al solubility in ReB_2_ with its inherently strong B-B and metal-B bonds and the mechanical properties of such solid solution thin films.

Herein, it will be demonstrated that Re_0.85_B_2_ as well as (Re_0.67_Al_0.10_)B_2_ and (Re_0.74_Al_0.11_)B_2_ thin films synthesized by RF-DC magnetron sputtering exhibit the ReB_2_ (*P6*_3_*/mmc*) structure. Additionally, an investigation of surface chemical reactions by X-ray photoelectron spectroscopy (XPS) will show that the Re_0.85_B_2_ thin film corroded upon exposure to atmosphere by forming perrhenic acid, whereas the (Re_0.74_Al_0.11_)B_2_ thin film formed a passivating Al-oxide layer suppressing a corrosive reaction. Furthermore, nanoindentation data will be compared to ab initio predictions of the elastic properties.

## Results and Discussion

**Figure 1:**
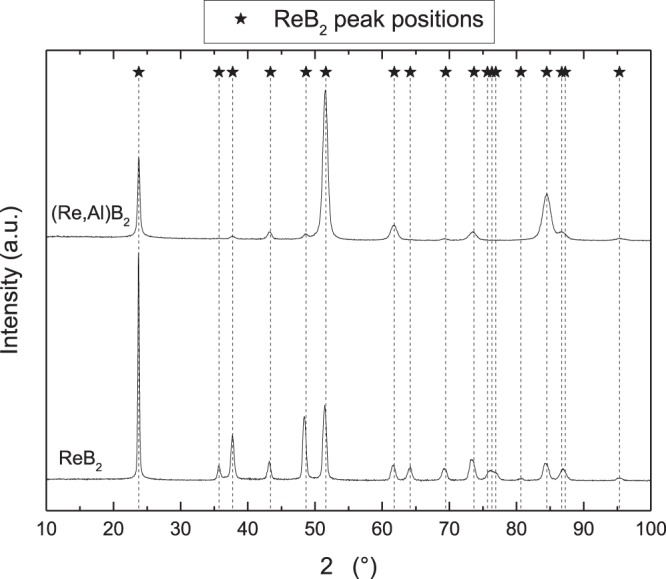
X-ray diffractogramms of studied samples. Shown are diffractograms of Re_0.85_B_2_ (bottom curve) and (Re_0.67_Al_0.10_)B_2_ (top curve) films. Peak positions of the ReB_2_ crystal structure (JCPDS card 00-11-5081) are indicated by markers with drop-lines.

Diffractograms of both Re_0.85_B_2_ and (Re_0.67_Al_0.10_)B_2_ films (Fig. 1., bottom and top curves, respectively) utilized for further mechanical characterization only display peaks attributable to the ReB_2_ crystal structure (JCPDS card 00-11-5081). No other Re-B phases or Al-borides are detected by XRD; hence, it is reasonable to assume that the 3.5 at.% Al in (Re_0.67_Al_0.10_)B_2_ is dissolved in the ReB_2_ crystal lattice and a solid solution is formed. While the Al concentration induced changes in lattices parameters measured by XRD (Δa = +0.31%, Δc = −0.31%) are opposite in trend to the values predicted by DFT (Δa = −0.08%, Δc = +0.25%), it has to be noted that the magnitude of the obtained deviations are according to Paier *et al*.^30^ in line with the exchange-correlation functionals employed here.

The films appear slightly B over-stoichiometric, potentially resulting in segregation of an amorphous B tissue phase^31^ or Re sub-lattice vacancies^32^. This is not further investigated here. Considering the measurement technology (energy dispersive X-ray spectroscopy) inherent errors which, according to Goldstein *et al*.^33^ can be larger than ±5% (relative) for rough samples and compounds containing light elements, the stoichiometric diboride composition lies within the error bars. For the chemical quantification, only intentionally introduced elements are considered here; however the samples contain C (ca. 4.5 at.%), O (ca. 1.0 at.%) and Fe (ca. 1.0 at.%) impurities, probably stemming from the B targets, residual gas contamination^34^, and RF sputtered chamber walls, respectively.

**Figure 2:**
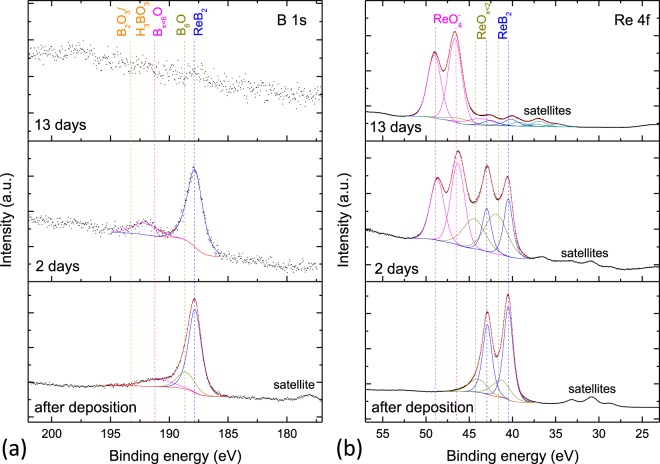
XPS scans of Re_0.85_B_2_. Shown are spectra of the (**a**) B 1 s and (**b**) Re 4 f transitions after different air exposure durations. The position of the components fitted to the spectra is indicated by drop-lines.

Surface chemical reactivity of Re_0.85_B_2_ in air was analyzed employing XPS measurements. The sample investigated here was transferred to the XPS system immediately after deposition. The initial air exposure time was less than 120 seconds. Subsequent measurements are conducted after exposing the sample to atmosphere for 2 days and 13 days. High resolution XPS spectra of the B 1 s and Re 4 f transitions are shown in Fig. 2(a,b), respectively. The bottom panels show the scans recorded right after the deposition. The minor signals that can be observed at lower binding energies next to the main signals are satellite peaks introduced by the non-monochromatic Al K_α_ radiation. The main B 1 s signal stemming from ReB_2_ is located at 187.9 eV. Information on the fitting of other components in the XPS spectra can be found in the supplementary material.

After two days in air, the lower peak-to-background ratio indicates a strongly decreased intensity of the B 1 s signal. A strong new component emerges at 46.5 eV in the Re 4 f scan, which can be assigned to the perrhenate ion (ReO_4_^−^)^26^. The ReO_4_^−^ ion is a constituent of Re_2_O_7_, which, when solved in water, forms perrhenic acid^35^. After 13 days in air, the Re 4 f signal consists almost completely of the ReO_4_^−^ component and the B 1 s signal has entirely disappeared. Over less than two weeks, the surface of Re_0.85_B_2_ becomes covered by several nanometers of perrhenic acid, taking into account that XPS depth resolution is in the order of a few nanometers and the B 1 s signal is undetectable. Orlovskaya *et al*.’s^23^ hypothesis about and Granados-Fitch *et al*.’s^24^ observation of formation of perrhenic acid during atmosphere exposure mechanically milled powders are confirmed here for thin films. There is no evidence for the formation of boric acid or ReO_3_ in this corrosion stage.

**Figure 3:**
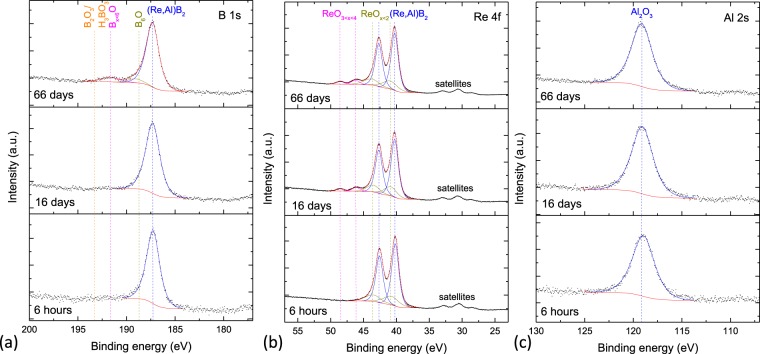
XPS scans of (Re_0.74_Al_0.11_)B_2._ Shown are spectra of the (**a**) B 1 s, (**b**) Re 4 f, and (**c**) Al 2 s transitions after different air exposure durations. The position of the components fitted to the spectra is indicated by drop-lines.

High resolution XPS spectra of the B 1 s, Re 4 f, and Al 2 s transitions of an (Re_0.74_Al_0.11_)B_2_ film after different air exposure durations are shown in Fig. 3(a–c), respectively. The Al 2 s transition is used instead of the more commonly used 2p transition because the latter is convoluted with the Re 4 f energy loss background. After approximately six hours in air, there is only one Al-oxide component in the Al 2 s signal located at 119.0 eV, which coincides excellently with literature values of Al_2_O_3_^36^. It should be noted that this means that the bonding environment and the resulting electron binding energy is comparable to that of Al_2_O_3_; it does not necessarily mean that the composition of the Al-oxide measured here is exactly that of Al_2_O_3_ and one cannot infer in which phase it is present. In the B 1 s scan, only the component of (Re,Al)B_2_ at 187.4 eV can be observed. The Re 4 f signal, similar to the unalloyed sample, shows the main (Re_0.74_Al_0.11_)B_2_ component located at 40.2 eV and a smaller component at 41.0 eV, which may originate from incipient oxidation or impurity bonds as discussed previously. After three days in air (measured but not shown in Fig. 3.), Al and B signals do not exhibit any change. A minor component located at higher binding energy (46.0 eV, labeled as ReO_3<x<4_ here), which exhibits an oxidation state between ReO_3_ and ReO_4_^−^, emerges in the Re 4 f scan. After 16 days in air (center panels in Fig. 3), this component increases only minimally from 4.2 to 5.1% of the total integrated intensity. Al and B spectra do still not show any change. After 66 days in air, small oxidized components (B_x<6_O, B_6_O) appear in the B 1 s spectrum; however, these components together make up only 13.7% of the total integrated intensity of the B signal, showing that B is not significantly oxidized after 66 days. The oxidized component in the Re 4 f spectrum, *i*, is increased to only 5.6% of the total integrated intensity after 66 days atmosphere exposure, so exhibiting approximately a 3.83 × *t*^0.09^ dependence, where *t* is the oxidation time in days. From a practical perspective, passivation of the film is achieved since *i* increases by only 0.05% from 4.71% after 9 days to 4.76% after 10 days of the film air exposure. Furthermore, no N is detected in this film. Initially, Re oxidizes a little while the Al-oxide layer is not fully evolved; nonetheless, the Al-oxide seems to passivate the film in the long term. Even if this passivation is mechanically destroyed (e.g. by wear), the Al-oxide layer will be restored since Al is incorporated in the ReB_2_ structure.

Hardness and elastic modulus measured by nanoindentation are given in Table 1. The Re_0.85_B_2_ film (*H*_*exp*_ = 32 GPa, *E*_*exp*_ = 522 GPa) does, on average, exhibit a hardness which is lower than the average of previously reported values (*H*_*lit*_ = 38(11) GPa^4,13–18^) and the elastic modulus is 21.7% lower than theoretically predicted (*E*_*DFT*_ = 667 GPa). The standard deviation on both quantities is with 25.7% relatively large. The (Re_0.67_Al_0.10_)B_2_ film, on the contrary, shows a higher hardness of 40 GPa, higher stiffness (*E* = 562 GPa, 7.7% higher than Re_0.85_B_2_), and lower standard deviations on the measured quantities. For both films, the indentation depth does not exceed the conventional 10% of the film thickness (Table 1); hence, a substrate influence is unlikely to explain the significant difference in measured mechanical properties. Instead, the measured surface roughness (Table 1) indicates that the Re_0.85_B_2_ film is much rougher than the (Re_0.67_Al_0.10_)B_2_ film. The Re_0.85_B_2_ film’s measured root mean square surface roughness of 38(1) nm is considerably large compared to the indenter tip radius of 100 nm. When indenting into a surface asperity, there is no continuous material on the sides opposing plastic deformation. The (Re_0.67_Al_0.10_)B_2_ film’s surface roughness of 18(2) nm lies below the microscope’s assumed resolution limit of 20 nm, suggesting 18 nm to be an upper limit of the surface roughness. The 100 nm radius indenter, therefore, indents into a continuous film rather than into surface asperities. This may contribute to the lower measured average and higher scattering of *H* and *E* of the rougher ReB_2_ film. The presence of porosity in the films may affect these quantities as well. A stark difference in surface roughness between the two films and indications for porosity in the Re_0.85_B_2_ film can be seen in SEM images in the supplementary information in Figs S1 and S2.

**Table 1:** Chemical composition and nanoindentation data for Re_0.85_B_2_ and (Re_0.67_Al_0.10_)B_2_ films.

Sample	at.% B	at.% Al	*H* (GPa)	*E* (GPa)	*E*_DFT_(GPa)	*h* (nm)	*D* (μm)	*R*_*q*_ (nm)
Re_0.85_B_2_	70.1	0.0	32(9)	522(134)	667	90(17)	1.52(6)	38(1)
(Re_0.67_Al_0.10_)B_2_	72.4	3.5	40(5)	562(56)	621	72(8)	1.83(9)	18(2)^a^

The columns display B and Al concentrations, average values of measured (standard deviation on the last significant digit is given in parenthesis) hardness, *H*, elastic modulus, *E*, indentation depth, *h*, film thickness, *D*, and root mean square surface roughness, *R*_*q*_.

^a^Resolution of the microscope is given as 20 nm. The stated value might overestimate the actual *R*_*q*_.

In summary, (Re_0.67_Al_0.10_)B_2_ and (Re_0.74_Al_0.11_)B_2_ solid solution and Re_0.85_B_2_ films were synthesized by hybrid RF-DC magnetron sputtering. All films crystalize in the ReB_2_ (*P6*_3_*/mmc*) crystal structure, as probed by XRD. Re_0.85_B_2_ reacts in humid air and forms perrhenic acid already after two days of atmosphere exposure, as shown by XPS. This corrosive reaction renders unalloyed ReB_2_ coatings unsuitable for application in humid air. Upon atmosphere exposure at room temperature (Re_0.74_Al_0.11_)B_2_, however, forms a self-passivating Al-oxide layer retarding the formation of perrhenic acid and, hence, corrosion. It is evident that Al additions to ReB_2_ significantly increase the chemical stability during atmosphere exposure.

## Methods

### Experimental details

(Re_0.67_Al_0.10_)B_2_, (Re_0.74_Al_0.11_)B_2_ and Re_0.85_B_2_ films were deposited by magnetron sputtering at 900 °C in a vacuum chamber accommodating four magnetrons (50 mm diameter) tilted to the substrate normal by 20° at a substrate-target distance of 12.4 cm. The average base pressure laid below 6.67 × 10^−5^ Pa (5 × 10^−7^ Torr). Ar was used as sputtering gas at constant working pressure of 0.67 Pa (5 × 10^−3^ Torr). The substrates’ backsides were coated with Mo for a better intake of radiative heat from the heater and, prior to deposition, the substrates were baked out for 20 minutes at deposition temperature. Re and Al targets were sputtered with direct current (DC) power supplies, while two B targets, facing each other, were sputtered by radio frequency (RF) power supplies.

Two Re_0.85_B_2_ samples were synthesized under identical conditions in the same batch (20 W at Re target, 2 × 148 W at B targets, 150 minutes) on polished MgO substrates at symmetry-equivalent positions on a rotating sample holder. The (Re_0.67_Al_0.10_)B_2_ sample was taken from a selected area of a combinatorial deposition (no rotation, 20 W at Re target, 7 W at Al target, 2 × 149 W at B targets, 150 minutes) on polished MgO. The (Re_0.74_Al_0.11_)B_2_ sample was taken from a selected area of another combinatorial deposition (no rotation, 15 W at Re target, 5 W at Al target, 2 × 150 W at B targets, 60 minutes) on polished sapphire.

The phase composition was analyzed by X-ray diffraction (XRD) employing Cu K_α_ radiation (U = 40 kV, I = 40 mA) at a constant incident angle ω = 10° and a 2θ range from 10° to 100° in a Bruker AXS D8 Discover General Area Detection Diffraction System (GADDS). Cross-sectional scanning electron microscope (SEM) images, taken in a FEI Helios 660 system, were employed for film thickness determination. Root mean square surface roughness was measured by confocal laser scanning microscopy in a Keyence VK-9700 system (resolution limit ca. 20 nm) on 1000 μm^2^ areas. Hardness and elastic modulus were investigated by nanoindentation with a 100 nm radius Berkovich diamond tip at 10 mN load in a depth-sensing nanoindenter (Hysitron TriboIndenterTM). 100 indentations were performed for each sample for sufficient statistics. A fused silica standard measured before and after the deposited films was used to calibrate the tip area function. Load-displacement curves exhibiting pop-in events (only one) were not considered for analysis. The Oliver-Pharr method^37^ was applied to obtain the reduced elastic modulus. A Poisson’s ratio of 0.18^38^ was assumed for the samples to calculate the samples’ elastic moduli.

The chemical composition of the films was quantified by energy dispersive X-ray spectroscopy (EDX) with an EDAX Genesis 2000 analyzer in a JEOL JSM-6480 SEM at an electron beam acceleration voltage of 5 kV. Chemical composition of the synthesized films was measured directly after the deposition and samples were stored in a high vacuum vessel (5.5 × 10^−5^ Pa base pressure) between all measurements due to the expected reactivity. One Re_0.85_B_2_ sample and the (Re_0.74_Al_0.11_)B_2_ sample were stored in air to evaluate their chemical stability. Chemical states at the surface of these two samples were investigated by XPS in a JEOL JAMP-9500F system with an Al K_α_ x-ray source (1486.5 eV), a hemispherical electron energy analyzer in fixed analyzer transmission mode with a pass energy of 20 eV, and an electron take-off angle perpendicular to the analyzer. The energy resolution is approximately 0.15 eV. For energy calibration, the Cu 2p 3/2 and 3p 3/2 lines (Ar^+^ cleaned) were employed. Charging of the sample was corrected against the C 1 s peak (284.8 eV) of adventitious carbon. Voigt functions and Shirley backgrounds were used for fitting the data.

### Computational details

Electronic structure calculations were used to calculate elastic properties of the pure ReB_2_ system and systems in which Re is randomly substituted by varying concentrations of Al. The systems considered contained 0.0, 3.1, and 8.6 at% of Al. Values of calculated lattice parameters and elastic moduli for the experimentally found compositions have been linearly interpolated. All supercells contained 162 atoms. The calculations were performed within the framework of density functional theory (DFT) employing the Vienna Ab initio Simulation Package (VASP)^39^. Projector-augmented wave potentials within PBE-GGA^40^ were used, for which the projector functions were evaluated in real space. Tetrahedron method smearing with Blöchl corrections^41^ was used with an electronic convergence criterion of 10^−4^ eV. All systems were structurally relaxed applying a conjugate-gradient algorithm with a force convergence criterion of 10^−2^ eV/Å. The equilibrium volume was found by a Birch-Murnaghan equation of state^42,43^ fit and the c/a-ratio was optimized by a third order polynomial fit. Cutoff energies of basis sets were equal to the distributor’s recommended value for the respective potential during dynamic relaxation and were further increased by 25% for static calculations. Integration in the Brillouin zone was performed on Γ-point-centered Monkhorst-Pack^44^ k-point grids converged to ≤1 meV per atom. Elastic constants were calculated by applying deformations to the cell geometry and fitting the change in total energy quadratically. Details were presented by Fast *et al*.^45^. Macroscopic elastic quantities (elastic modulus and Poission’s ratio) were calculated from elastic constants using the Hill (Reuss-Voigt-average) method^46^.

All data generated or analyzed during this study are included in this published article and its supplementary information files.

## Electronic supplementary material

Supplementary Information
