# Supplementary material for: Self-passivating (Re,Al)B2 coatings synthesized by magnetron sputtering
Source: Sci Rep. 2018 Oct 22;8:15570. doi: 10.1038/s41598-018-34042-1 (PMC6197250; doi:10.1038/s41598-018-34042-1)
Supplement: Supplementary file 1 — Supplementary Information [file 41598_2018_34042_MOESM1_ESM.pdf]

Supplementary information to "Self-passivating (Re,Al)B_2 coatings synthesized by magnetron sputtering"

Pascal Bliem*, Stanislav Mráz, Sandipan Sen, Oliver Hunold, and Jochen M. Schneider

Additional information on the fitting of XPS spectra:

In Fig. 2., right after the deposition, there are two more components that can be fitted to the scan, of which one can be assigned to B_6O[1] at 188.7 eV. The other component represents a higher oxidation state (labeled as $B_{x<6}O$ here), which is, however, not located at a high enough binding energy to be assigned to B_2O_3 or H_3BO_3[1]. The 7/2 peak of the strong Re 4f doublet from $Re_{0.85}B_2$ is located at 40.5 eV. There is another doublet component at higher binding energies (41.4 eV, labeled as $ReO_{x<2}$ here), which can be fitted to the Re 4f signal. This component's binding energy is too low to be attributed even to the lowest Re oxide (ReO_2 at 43.0(5) eV[1]). Hence, it is unclear whether the component truly represents the onset of oxidation or stems from bonds with Fe or C impurities, frequent lattice defects, or even phase impurities undetectable by XRD. These aspects may simultaneously contribute to the component considering that the peak is relatively broad. After two days in air, the $B_{x<6}O$ component increases its oxidation state as it migrates to higher binding energies; yet it can still not be matched to B_2O_3 or H_3BO_3. Furthermore, the $Re_{0.85}B_2$ film seems to incorporate N from the air (N spectrum is not shown here). There is no N detected immediately after the deposition; however, the N concentration increases with increasing air exposure. The breaking of molecular N_2 bonds (and probably reduction of N_2) can be an indicator for an increased catalytic activity of the film surface[2]. This catalytic activity may accelerate the corrosive reaction.

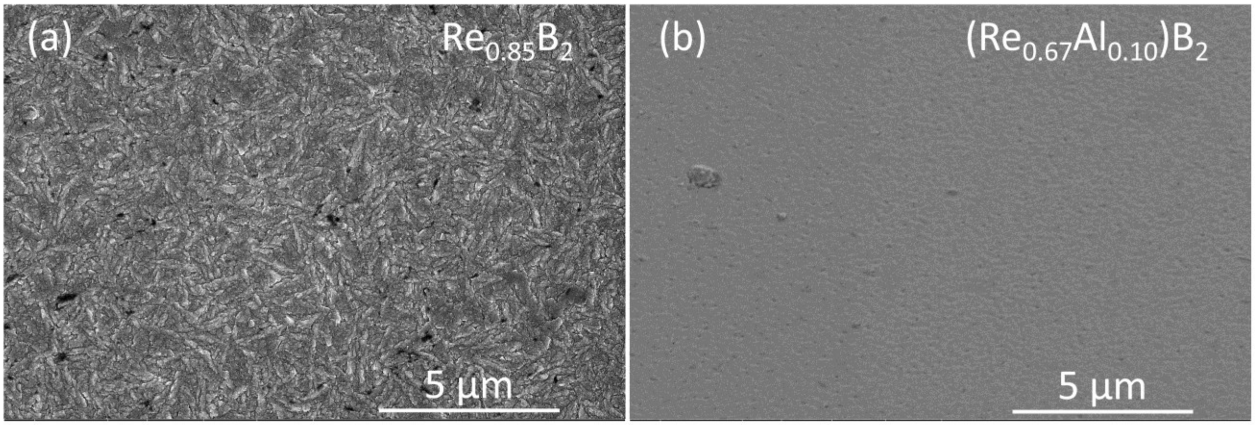

Figure S1. Top view scanning electron microscope images. Shown are top views of (a) $Re_{0.85}B_2$ and (b) $(Re_{0.67}Al_{0.10})B_2$ films. The roughness of $Re_{0.85}B_2$ is clearly higher than that of $(Re_{0.67}Al_{0.10})B_2$ and the dark spots in (a) are likely to be pores, which explains the large difference in hardness and stiffness between the two films.

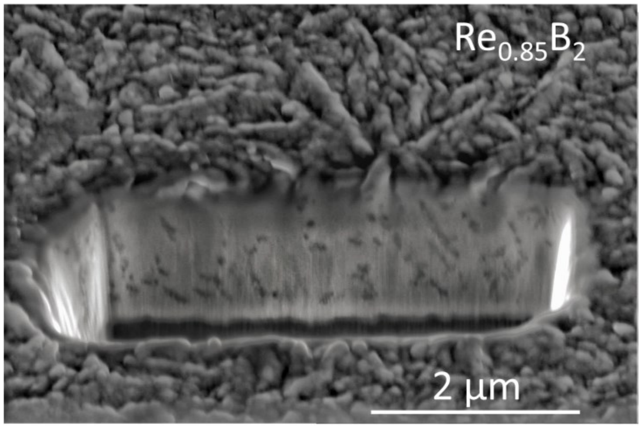

Figure S2. Cross-section scanning electron microscope image of the $Re_{0.85}B_2$ film. The darker regions in the cross-section are likely to be a manifestation of porosity present throughout the entire film thickness.

References

1. National Institute of Standards and Technology, NIST X-ray Photoelectron Spectroscopy Database, Version 4.1., Gaithersburg MD, 20899 (2000)
2. Shilov, A. E. Catalytic reduction of molecular nitrogen in solutions. *Russ. Chem. Bull.* **52**, 2555–2562 (2003).
